# Translating intracarotid artery transplantation of bone marrow‐derived NCS‐01 cells for ischemic stroke: Behavioral and histological readouts and mechanistic insights into stem cell therapy

**DOI:** 10.1002/sctm.19-0229

**Published:** 2019-11-18

**Authors:** Yuji Kaneko, Jea‐Young Lee, Naoki Tajiri, Julian P. Tuazon, Trenton Lippert, Eleonora Russo, Seong‐Jin Yu, Brooke Bonsack, Sydney Corey, Alexandreya B. Coats, Chase Kingsbury, Thomas N. Chase, Minako Koga, Cesar V. Borlongan

**Affiliations:** ^1^ Center of Excellence for Aging and Brain Repair, Department of Neurosurgery and Brain Repair University of South Florida College of Medicine Tampa Florida; ^2^ KM Pharmaceutical Consulting LLC Washington District of Columbia

**Keywords:** cell loss, cell transplantation, cerebral ischemia, cytokines, functional recovery, infarct, motor deficits

## Abstract

The present study used in vitro and in vivo stroke models to demonstrate the safety, efficacy, and mechanism of action of adult human bone marrow‐derived NCS‐01 cells. Coculture with NCS‐01 cells protected primary rat cortical cells or human neural progenitor cells from oxygen glucose deprivation. Adult rats that were subjected to middle cerebral artery occlusion, transiently or permanently, and subsequently received intracarotid artery or intravenous transplants of NCS‐01 cells displayed dose‐dependent improvements in motor and neurological behaviors, and reductions in infarct area and peri‐infarct cell loss, much better than intravenous administration. The optimal dose was 7.5 × 10^6^ cells/mL when delivered via the intracarotid artery within 3 days poststroke, although therapeutic effects persisted even when administered at 1 week after stroke. Compared with other mesenchymal stem cells, NCS‐01 cells ameliorated both the structural and functional deficits after stroke through a broad therapeutic window. NCS‐01 cells secreted therapeutic molecules, such as basic fibroblast growth factor and interleukin‐6, but equally importantly we observed for the first time the formation of filopodia by NCS‐01 cells under stroke conditions, characterized by cadherin‐positive processes extending from the stem cells toward the ischemic cells. Collectively, the present efficacy readouts and the novel filopodia‐mediated mechanism of action provide solid lab‐to‐clinic evidence supporting the use of NCS‐01 cells for treatment of stroke in the clinical setting.

## Graphical abstract

NCS‐01 cell therapy for stroke. Stroke models of oxygen glucose deprivation and middle cerebral artery occlusion reveal typical cell death depicting clinically relevant stroke pathology. Coculture or transplantation of NCS‐01 cells attenuate stroke‐induced pathological deficits as evidenced by increased cell viability and mitochondrial activity in cultured primary rat cortical cells and decreased infarct area and peri‐infarct cell loss in the rat stroke brain. Potential mechanisms of action include upregulation of bFGF and IL‐6 coupled with filopodia formation.

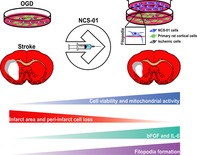

**Significance statement**

The present study recognizes critical translational gaps in stem cell transplant dose, route, and timing after stroke, and acknowledges solid safety profile of mesenchymal stem cells. The study tested a human bone marrow‐derived mesenchymal stem cell line called NCS‐01 in oxygen glucose deprivation and middle cerebral artery occlusion models, which revealed the optimal dose of 7.5 × 106 cells/mL via the intracarotid artery within 3 days poststroke. Secretion of cytokines, specifically bFGF and IL‐6, and filopodia formation, are potential mechanisms. Based on these preclinical data, the FDA in July 2019 approved intracarotid NCS‐01 cell transplantation in ischemic stroke patients.

## 1 INTRODUCTION

Stroke remains as one of the most prevalent causes of disability and death among adult populations around the world,^1^ significantly costing the United States billions of dollars each year.^2^ Tissue plasminogen activator (tPA) is the sole FDA‐approved drug to treat acute ischemic stroke, which accounts for roughly 87% of all strokes.^3^, ^4^ tPA is most effective when administered intravenously (IV) within 4.5 hours of stroke onset,^4^ but is toxic outside this therapeutic window, causing hemorrhagic transformation.^4^ Mechanical thrombectomy serves as an alternative treatment for ischemic stroke, but it too encounters challenges such as a limited therapeutic window (6‐24 hours post stroke),^5^ bleeding, coagulation abnormalities, and intracranial hemorrhage.^6^ Since most stroke patients do not have access to tPA therapy or qualify for mechanical thrombectomy within the limited therapeutic windows, novel treatments are warranted. Cell‐based regenerative medicine has emerged as a safe and effective experimental treatment for stroke and has reached clinical trials.

The central nervous system has long been considered as incapable of regeneration. Stem cell research has challenged this paradigm with compelling evidence of exogenous and endogenous repair processes.^7^ Transplantation of embryonic, fetal, umbilical, amnion, and induced pluripotent stem cells shows functional improvements in experimental stroke.^8^, ^9^ Adult bone marrow‐derived stem cells, such as endothelial progenitor cells, hematopoietic, and mesenchymal stem cells (MSCs), have expedited the translation of lab‐to‐clinic stem cell therapy due to their logistical ease in isolation and amplification, and being relatively free from ethical concerns.^10^, ^11^ MSCs have been explored as transplantable donor cells for many experimental models of neurological diseases,^12^, ^13^ such as Parkinson's disease,^14^, ^15^, ^16^ amyotrophic lateral sclerosis,^17^, ^18^, ^19^ Alzheimer's disease,^20^, ^21^ and stroke.^22^, ^23^, ^24^, ^25^, ^26^, ^27^, ^28^ Cell replacement was initially implicated in MSCs' therapeutic effects, yet with only modest graft survival despite robust functional outcomes,^29^ the currently accepted mechanism of action involves bystander repair processes primarily via stem cell‐secreted therapeutic factors.^30^, ^31^, ^32^, ^33^ This promising preclinical evidence, as well as solid safety record in treating hematological diseases, provides concrete grounds for clinical translation of MSC therapy in stroke. However, two clinical trials examining MSCs upheld their safety, but did not reveal efficacy.^34^, ^35^ IV transplantation of autologous bone marrow MSCs at 4 weeks following stroke reputedly demonstrated enhanced neurological outcomes, yet these functional improvements diminished by 12 months after transplantation.^34^ An inconsistent adherence may have contributed to strict doses and therapeutic time windows. For example, preclinical investigations employ approximately 4 million cells for IV administration for a 250 g stroke rat, corresponding to 840 million cells in a human of 75 kg, whereas clinical trials have employed doses well below those deemed optimal in preclinical animal models.^34^, ^35^ This lack of translation of optimal laboratory parameters may explain these failed clinical efforts despite overwhelming experimental evidence.^23^ Recognizing these critical translational gaps, while acknowledging the long‐standing safety profile and the abundance of preclinical studies demonstrating neuroprotective effects, provides a solid rationale for testing the efficacy of an MSC line toward advancing stem cell therapy in stroke.

In identifying transplantable bone marrow‐derived MSCs for clinical application, we used lab‐to‐clinic translational research criteria, namely the cells need to be of human origin, clinical grade, ample supply, and with well‐defined phenotypic markers. To this end, the adult bone marrow‐derived NCS‐01 cells satisfy all these criteria. From the basic science research standpoint, transparency of the phenotypic characteristics of NCS‐01 cells should allow the cells' reproducibility and direct comparisons with other MSC transplantation studies. From the clinical research view, the human, clinical grade and ample supply should aid in optimization of the envisioned human clinical product and circumvent inherent translational problems when testing rodent MSCs as transplant material. Accordingly, the present study employed highly translational stroke paradigms to evaluate the safety, efficacy, and mechanism of action of NCS‐01 cells, using the in vitro oxygen glucose deprivation (OGD) model, and the in vivo transient and permanent middle cerebral artery occlusion (MCAO) models. Optimal route of administration, and a side‐by‐side comparison with the current standard MSCs, allowed us to directly assess the therapeutic effects of NCS‐01 cells in ameliorating stroke‐induced behavioral and histological deficits, eventually serving as a guide for the envisioned clinical trial design. Based on the preclinical data presented here, we recently received in early July 2019 the FDA's approval to proceed with the clinical application of intracarotid artery (ICA) transplantation of NCS‐01 cells in ischemic stroke patients.

## 2 METHODS

The data that support the findings of this study are available from the corresponding author upon reasonable request. This series of highly translational studies utilized NCS‐01 cells, which are human bone marrow‐derived MSCs produced by Progenitor Cell Therapy (Mountain View, California). The release criteria included phenotypic characterization of these cells via fluorescence‐activated cell sorting, indicating that these cells are CD105+, CD73+, CD90+, CD34−, CD45−, and CD14−. Subsequent release criterion added the capacity of these cells to secrete high amounts of basic fibroblast growth factor (bFGF) and interleukin‐6 (IL‐6). Cell viability of NCS‐01 cells was confirmed at least >85% prior to starting each experiment. Engraftment was also confirmed for each transplant experiment and revealed modest graft survival, with graft persistence almost nondetectable by day 3 post‐transplantation (Supplemental Figure S1). No ectopic tissue or tumor formation was detected in any study.

### 2.1 In vitro studies

#### 2.1.1 Cell culture

Mixed cultures of gestation age E18 primary cortical neurons and astrocytes were initially used as host cells (CAMBREX, Maryland). To further enhance the translational relevance of the study, subsequent cell culture studies used human neural progenitor cells (Neuromics, Maryland) to better mimic the human condition. Immediately after thawing, cells (4 × 10^4^ cells/well) were seeded and grown in 96‐well plate coated by poly‐L‐lysine in Neurobasal media (GIBCO, California) containing 2 mM L‐glutamine, 2% B27 (GIBCO) and 50 U/mL penicillin and streptomycin for 7‐10 days at 37°C in humidified atmosphere containing 5% CO_2_. To further probe the mechanism of action of NCS‐01 cells, a vis‐a‐vis in vitro efficacy test was performed comparing the targeted cell viability and NCS‐01 cells' filopodia formation when cocultured with a specific neural cell lineage, including primary rat cortical neurons (CAMBREX), primary rat astrocytes (Fisher), and primary rat endothelial progenitor cells (EPCs) (Harvard, Massachusetts). Neurons were seeded and grown in culture as above. Astrocytes were seeded and grown in high glucose DMEM (Fisher), 10% fetal bovine serum (Neuromics), and 1% penicillin and streptomycin. EPCs were seeded and grown in endothelial cell growth basal medium‐2 (Lonza, CH), 5% fetal bovine serum, 0.2 mL hydrocortisone, 2 mL human bFGF, 0.5 mL vascular endothelial growth factor (VEGF), 0.5 mL R3 insulin‐like growth factor‐1 (IGF‐1), 0.5 mL ascorbic acid, 0.5 mL human epidermal growth factor, and 0.5 mL gentamicin‐amphotericin‐B (GA‐1000) (Lonza, CH). Neurons, astrocytes and EPCs were likewise seeded and grown in a 24‐well plate (coated with poly‐D‐lysine for neurons only) for 7‐10 days at 37°C in humidified atmosphere containing 5% CO_2_.

#### 2.1.2 OGD model

The cultured primary rat cortical cells or human neural progenitor cells were exposed to OGD as described previously^36^ with few modifications. Briefly, the culture medium was replaced with a glucose‐free Earle's balanced salt solution with the following composition: 116 mM NaCl, 5.4 mM KCl, 0.8 mM MgSO_4_, 1 mM NaH_2_PO_4_, 26.2 mM NaHCO_3_, 0.01 mM glycine, 1.8 mM CaCl_2_, and pH adjusted to 7.4. Cultured cells were placed in humidified chamber, and then equilibrated with continuous flow of 95% N_2_ and 5% CO_2_ gas for 15 minutes. After this equilibrium, the chamber was sealed and placed into the incubator at 37°C for 90 minutes. Thereafter, OGD was terminated by adding glucose to the culture medium and returning the cultures to the standard 20% O_2_ and 5% CO_2_ incubator. A 1‐ or 2‐hour period of “reperfusion” in standard medium and normoxic condition was allowed.

#### 2.1.3 Cell survival

The viability of primary rat cortical cells or human neural progenitor cells was evaluated immediately after OGD (no NCS‐01 cells added) and at 5 hours after OGD using standard 3‐(4,5‐dimethylthiazolyl‐2)‐2,5‐diphenyltetrazolium bromide (MTT) and trypan blue exclusion assays. Following reperfusion, reduction of MTT by cellular dehydrogenases was used as a measure of mitochondrial activity as previously described.^37^ In addition, trypan blue (0.2%) exclusion method was conducted and mean viable cell counts were calculated in four randomly selected areas (1 mm^2^, n = 10) to reveal the cell viability after the ischemic‐reperfusion condition. Briefly, within 5 min after adding trypan blue, we digitally captured under microscope (×200) 10 pictures (approximately 100 cells per picture) for each condition, then randomly selected 5 pictures, and counted the number of cells for each individual treatment condition. Normalized cell viability was calculated from the following equation: viable cells (%) = [1.00 − (Number of blue cells / Number of total cells)] × 100.^38^

#### 2.1.4 Cytokine analysis

Cytokines were measured in the supernatant of cultured cells at 5 hours after OGD using commercially available ELISA kits to detect initially human brain derived neurotrophic factor (BDNF), beta‐nerve growth factor (β‐NGF), IGF‐1, VEGF, bFGF, and IL‐6, but subsequently just focused on bFGF, and IL‐6, which were chosen based on our pilot studies demonstrating that the expression levels of these two cytokines were consistently upregulated in the supernatant of cultured NCS‐01 cells. Briefly, the supernatant was collected from the culture medium of NCS‐01 cells and transferred into a centrifuge tube which was then centrifuged at 1500 rpm for 10 minutes at 4°C. Thereafter, aliquots of the supernatant were collected and stored at −80°C until use. Concentrations of cytokines were detected using ELISA kits (Abcam, United Kingdom) according to the manufacturer's instructions.

#### 2.1.5 Filopodia formation assay and IL‐6 and bFGF treatment

This set of experiment was designed to reveal a potential regenerative mechanism mediating the therapeutic effects of NCS‐01 cells on ischemic cells. Primary rat cortical neurons (which may also include a small percentage of glial cells) were subjected to OGD‐reperfusion paradigm as described above. Next, NCS‐01 cells were cocultured with the host cells for 3 hours at 37°C using a two‐chamber system (Fisher), with NCS‐01 cells suspended on the upper chambers at different distances (ranging from 0 to 2.04 mm) above host cells which were seeded on the lower chambers containing DMEM medium and poly‐L‐lysine coated glass coverslips. The lower chambers that contained host rat cells only (since the upper chambers small pore size did not allow migration of human NCS‐01 cells toward the lower chambers, as confirmed by lack of HuMito stained cells in these chambers) were then processed for immunocytochemical analyses of formation of human filopodia using N‐cadherin antibodies (Abcam). Double labeling with a neuronal cell death marker using propidium iodide (Abcam) staining was also carried out to assess any interaction between NCS‐01 cell‐derived filopodia formation and primary rat cortical neuron viability. A control group was also established comprising cortical neurons that were not subjected to OGD nor received any treatment. In the additional mechanistic probe test, primary rat cortical neurons, primary rat astrocytes, and primary rat EPCs were subjected to OGD and reperfusion as described above. 1 ng/mL each of IL‐6 and bFGF was administered to half of the host cells after undergoing OGD and reperfusion, such that one group received cell media only (OGD only control), another group received IL‐6 + bFGF, a third group received the NCS‐01 cell coculture at a distance of 0.8 mm, and a final group received IL‐6 + bFGF and the NCS‐01 cell coculture at a distance of 0.8 mm. After 12 hours at 37°C, MTT assay and N‐cadherin staining were performed.

### 2.2 In vivo studies

#### 2.2.1 Subjects

All experimental procedures were approved by the University of South Florida Institutional Animal Care and Use Committee. Male Sprague‐Dawley rats (8 weeks old, n = 3‐10 per treatment group) were housed under normal conditions, with two animals housed per cage (20°C temperature, 50% relative humidity, and 12‐hour light/dark cycle). The rats had free access to water and food. All necessary precautions were taken in order to reduce pain and suffering of the animals throughout the study. Animals were closely checked twice per day. All studies were performed by personnel blinded to the treatment condition.

#### 2.2.2 MCAO model

As previously described, stroke surgery was performed using the MCAO technique.^28^, ^39^, ^40^ Animals were anesthetized with a mixture of 1% to 2% isoflurane in nitric oxide/oxygen (69%/30%) via a face mask, and body temperature was maintained at 37 ± 0.3°C during the surgical procedures. A midline skin incision was made in the neck with subsequent isolation of the left common carotid artery, the external carotid artery (ECA), and internal carotid artery. Thereafter, a 4‐0 monofilament nylon suture (15.0‐17.0 mm) was advanced from the common carotid artery bifurcation until it blocked the origin of the MCA. The skin incision was closed with surgical clips. Animals were allowed to recover from anesthesia during MCAO. At 1 hour after MCAO, animals were re‐anesthetized, and reperfusion commenced with the withdrawal of the nylon suture. We have standardized the MCAO model, with stroke animals showing ≥80% reduction in regional cerebral blood flow (CBF) during the occlusion period as determined by laser Doppler (Perimed, Periflux System 5000). For baseline regional CBF measurement, the laser Doppler probe was placed over the right frontoparietal cortical area supplied by the MCA. We also found no significant differences in physiological parameters, including PaO_2_, PaCO_2_, and plasma pH measurements. Rats that reached the 80% regional CBF reduction and >75% biased swing activity (see below) during occlusion were enrolled in these studies. Thereafter, incisions were closed, and animals were allowed to recover from anesthesia. Whereas for transient MCAO, the nylon suture blocked the MCA for 60 minutes, for permanent MCAO, the nylon suture was not removed.

#### 2.2.3 Treatment

At specified time points after MCAO, animals were randomly assigned to receive ICA (via the internal carotid artery) transplants of either saline/fibroblast cells (placebo) or NCS‐01 cells. Since the animals received right MCAO, animals also received infusion of saline/fibroblast cells or stem cells into the right ICA. Our ipsilateral ICA procedure also allowed infusion of saline/fibroblast cells and NCS‐01 cells in permanent MCAO. Cell doses were based on pilot studies that showed efficacy without adverse effects (ie, microembolism). Based on our experience, efficacy with ICA may be obtained at a lower dose of stem cells compared to IV route of delivery. Infusion of stem cells was performed via an autoinjection pump (Harvard Apparatus) at a rate of 1 mL/min. A PE‐10 polyethylene catheter connected to a 25G butterfly needle and a 1‐mL syringe was inserted into the stump of right ECA through an incision on the vessel and was tightened to the ECA by sutures. The catheter was positioned forward into ICA and passed the pterygopalatine artery, the extracranial branch of ICA, in order to enhance the delivery of the saline/fibroblast cells or stem cells toward the brain. The catheter was filled with saline/fibroblast cells or stem cells to prevent formation of potential harmful air bubble. The syringe was also filled with saline/fibroblast cells or stem cells. After dosing, the needle was removed without any flush and the CCA was closed with sutures to prevent any bleeding, and the skin wound reclosed with surgical clips. NCS‐01 cell doses ranged from 7.5 × 10^4^ to 3.75 × 10^7^ total in 0.1 to 5 mL with 7.5 × 10^6^ cell/mL concentration. In subsequent studies, the ICA route was vis‐à‐vis compared with IV administration (via the jugular vein).

#### 2.2.4 Behavioral tests

All investigators testing the animals were blinded to the treatment condition. Each rat was subjected to a series of behavioral tests to reveal motor and neurological performances at different time points before and after stroke and transplantation of NCS‐01 cells. The tests included the modified Bederson neurological test (composite score of contralateral hind limb retraction, beam walking ability, and bilateral forepaw grasp), while motor function was assessed by the Elevated Body Swing Test (EBST). The Bederson neurological test is an evaluation of the animal's sensorimotor function, which consists of three distinctive phases, performed over approximately 10 minutes per rat. Each phase of the test was conducted sequentially and was each scored 0 to 3. These three evaluations were: 1. contralateral hind limb retraction; 2. bilateral forepaw grasp; and 3. beam walking ability. Contralateral hind limb retraction measured the ability of the animal to replace the hind limb after it was displaced laterally by 2 to 3 cm. Grades were as follows: 0 for immediate smooth replacement, 1 for slow replacement, 2 for partial rigid replacement, and 3 for no replacement. Bilateral forepaw grasp measured the ability of a rat to hold onto a 2‐mm diameter steel rod. Grade 0 was used for rats with normal forepaw grasping behavior, 1 for rapid grasping but with rigidity, 2 for slow grasping with rigidity, and 3 for a rat unable to grasp with the forepaw. Beam walking ability used a beam apparatus that was 80 cm long with a flat surface of 2‐4 cm width resting at least 40 cm above the table/surface top on two poles. The animal was placed at one end of the beam then the ability to traverse the beam and reach the other end was assessed. The grades were as follows: 0 for a rat that easily traversed the beam, 1 if the rat slowly traversed the beam, 2 for partially traversing the beam but falls off, and 3 for a rat unable to stay on the beam for 10 seconds. The scores from all three tests were added to give a total neurologic deficit score (maximum possible score is 9 with mean composite neurologic score of 3). A score of 2.5 was set as a criterion to be considered a “stroke” animal. EBST is a measure of asymmetrical motor behavior that does not require animal training or drug injection.^41^ The rats were held, in the vertical axis, approximately 1 in from the base of its tail and then elevated to an inch above the surface on which it has been resting. The frequency and direction of the swing behavior were recorded for over 20 tail elevations. A swing was counted when the head of the rat moved more than 10° from the vertical axis to either side. Normally, intact rats display a 50% swing bias, that is, the same number of swings to the left and to the right. A 75% swing bias toward one direction was used as criterion of motor deficit.^41^ The total number of swings made to the biased side was added per group and divided by the n, providing the average number of swings per treatment group.

#### 2.2.5 Euthanasia and perfusion

Under deep anesthesia, rats were euthanized for immunofluorescent and protein analysis. For immunofluorescent analysis, briefly, animals were perfused through the ascending aorta with 200 mL of cold PBS, followed by 200 mL of 4% paraformaldehyde in phosphate buffer (PB). Brains were harvested and postfixed in the same fixative for 72 hours, followed by 30% sucrose in PB until completely sunk. Series of coronal sections were cut at a thickness of 40 μm using a cryostat and stored at −20°C. Brains were harvested and coronal sections were collected at a thickness of 2 mm using a brain matrix.

Staining for human mitochondria‐positive cells was conducted on every 1 of 6 sections, 40 mm thick in brain. All sections were washed three times for 5 minutes in PBS. Sections were incubated with saline sodium citrate solution at pH 6 for 40 minutes at 80°C for antigen retrieval. Next, samples were blocked for 60 minutes at room temperature with 8% normal goat serum (Invitrogen, California) in PBS containing 0.1% Tween 20 (PBST; Sigma). Sections were then incubated overnight at 4°C with mouse anti‐HuMito (Abcam) with 4% normal goat serum. Thereafter, the sections were washed five times for 10 minutes in PBST and soaked in 4% normal goat serum in PBST containing corresponding secondary antibodies, goat anti‐rabbit IgG‐Alexa Fluor 594 (red; 1:500; ThermoFisher Scientific) for 90 minutes. Finally, sections were washed five times for 10 minutes in PBST and three times for 5 minutes in PBS, cover‐slipped with Vectashield hardset with DAPI (H‐1500, Vector Laboratories, California). All sections were examined using a confocal microscope (Zeiss). Control studies included exclusion of primary antibody substituted with 5% normal goat serum in PBS. No immunoreactivity was observed in these controls.

#### 2.2.6 Histology

Alternate brain tissue sections were processed for Nissl staining, which was performed with 0.1% cresyl violet solution (Sigma‐Aldrich) using a standard protocol to evaluate the peri‐infarct injury of our MCAO model. From each perfused brain, six coronal sections between the anterior edge and posterior edge of the MCAO infarct area were collected and processed for Nissl staining. Every sixth coronal tissue section was chosen at random to quantify cell survival in the peri‐infarct area. Brain sections were examined using a light microscope (Keyence). Neuronal survival in the peri‐infarct area of the brain was quantified using a computer‐assisted image analysis system (NIH Image Software) and was expressed as a percentage of the ipsilateral hemisphere compared to the contralateral hemisphere.

#### 2.2.7 Statistical analyses

The data were evaluated statistically using two‐way ANOVA followed by post hoc Bonferroni corrected pairwise comparison *t*‐tests. Statistical significance was preset at *P* < .05 (Statview). The Kolmogorov‐Smirnov test was performed to assess normality and the resulting values were less than 5% of the critical values.

## 3 RESULTS

### 3.1 Study 1

#### 3.1.1 Higher doses of NCS‐01 cells increase primary rat cortical cell viability

Dosage evaluation is necessary to determine optimal safety and efficacy of NCS‐01 cells. Dose‐response in vitro studies entailed adding different doses of NCS‐01 cells to primary rat cortical neurons and astrocytes (host cells) after 2‐hour reperfusion, with dosages expressed as percent ratio of NCS‐01 cells to host cells. Each well contained 4 × 10^4^ host cells. Doses of 1 × 10^4^, 2 × 10^4^, 3 × 10^4^, 4 × 10^4^, 8 × 10^4^, or 1.6 × 10^5^ cells/well, equating to 25%, 50%, 75%, 100%, 200%, or 400% of host cell numbers, were used and compared to contemporaneous OGD controls with 0% NCS‐01 cells. An additional low dosage of 2 × 10^3^ cells/well or 5% of host cell numbers was subsequently assessed. Cell viability of host cells was assessed immediately after OGD and at 5 hours after OGD. Trypan blue was used as an indicator of host cell death while MTT assay assessed metabolic or mitochondrial activity of the cells.

**Figure 1:**
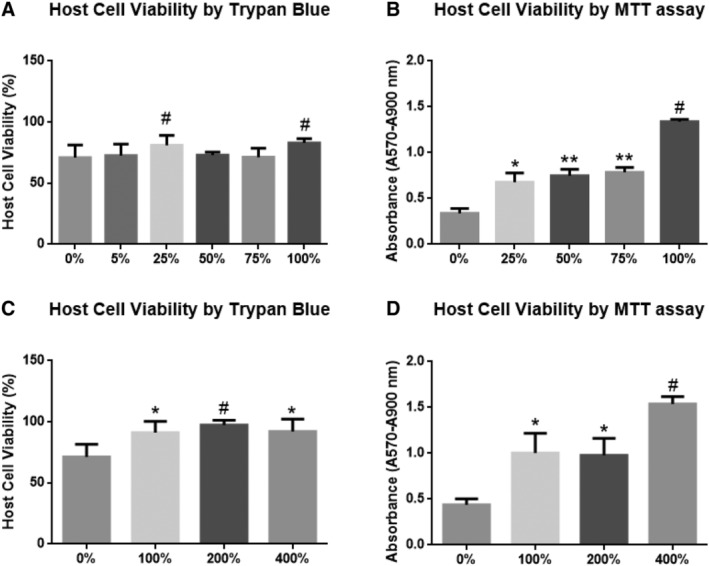
NCS‐01 cells protect cultured primary rat cortical cells against oxygen glucose deprivation (OGD). Host cell viability was measured at different dosages relative to the number of host cells. Cell survival was assessed via trypan blue for control (0%) and at NCS‐01 cell amounts ranging from 5% to 100%. Ratios of 1:4 and 1:1 displayed the greatest survival (#*P* < .05 vs 0%) (A). Cell survival was also assessed via trypan blue for control (0%) and higher amounts of NCS‐01 cells, ranging from 100% to 400% (*P < .01, #*P* < .001 vs 0%) (C). All three of the higher ratios exhibited robust survival in comparison to the control, *P < .01. Mitochondrial activity was measured via MTT assay for the control and for NCS‐01 cell amounts ranging from 25% to 100% (*P < .01, **P < .001, #*P* < .0001 vs 0%) (B) and from 100% to 400% (*P < .05, #*P* < .0001 vs 0%) (D), with another control. All ratios improved absorbance, with 4:1 providing the best results

NCS‐01 cells rescued against host cell death in direct relation to the number of NCS‐01 cells added (Figure 1). When measured by trypan blue (Figure 1A,C), 5% NCS‐01 cell wells exhibited no appreciable difference over the OGD control. Twenty‐five percent of NCS‐01 cell wells minimally rescued against host cell death compared to the OGD control (*P* < .05). Fifty percent and 75% NCS‐01 cell wells showed modest, nonsignificant increases in cell viability over the OGD control (*P* > .05). Ratios of 1:1, 2:1, and 4:1 of NCS‐01 cells to host cells produced the largest increases in rescue of host cell death, with all at least 20% greater than their OGD controls (*P* < .05, *P* < .01, *P* < .0001) (Figure 1C). No significant difference was observed between these three higher doses. The greatest increase was found in the 200% NCS‐01 cell wells, which were approximately 35% greater than the OGD control (*P* < .0001). In addition, MTT assay revealed a similar pattern (Figure 1B,D). Dosages of 25%, 50%, 75%, 100%, and 200% NCS‐01 cells produced approximately onefold increments in MTT absorbance over 0% NCS‐01 cells or OGD controls (*P* < .01, *P* < .001, *P* < .0001). The 400% NCS‐01 cell wells exhibited the most benefit, displaying a twofold increase in mitochondrial activity over the OGD control (*P* < .0001).

Based on these in vitro findings, NCS‐01 cells exerted host cell death rescue as assessed by trypan blue and MTT assays. Results suggest that a significantly robust therapeutic effect required at least a 1:1 ratio of NCS‐01 cells to primary rat cortical neurons and astrocytes. Collectively, these promising results of the in vitro dose‐response experiments provided the impetus to examine NCS‐01 cells under in vivo stroke paradigms.

#### 3.1.2 Route type favors ICA over IV cell delivery for reducing infarct area, but both routes comparably improve neurological function

Based on the above in vitro dose finding study, in vivo experiments were conducted to find the best route to most effectively deliver NCS‐01 cells into the target lesion. This study compared ICA and IV routes in a 2 × 2 between groups design. Groups of three (for IV administration) or six (for ICA administration) male rats were subjected to 1 hour transient MCAO and were treated with either saline or 7.5 × 10^6^ NCS‐01 cells in 1 mL. Animals were followed‐up for up to 7 days post‐transplantation.

**Figure 2:**
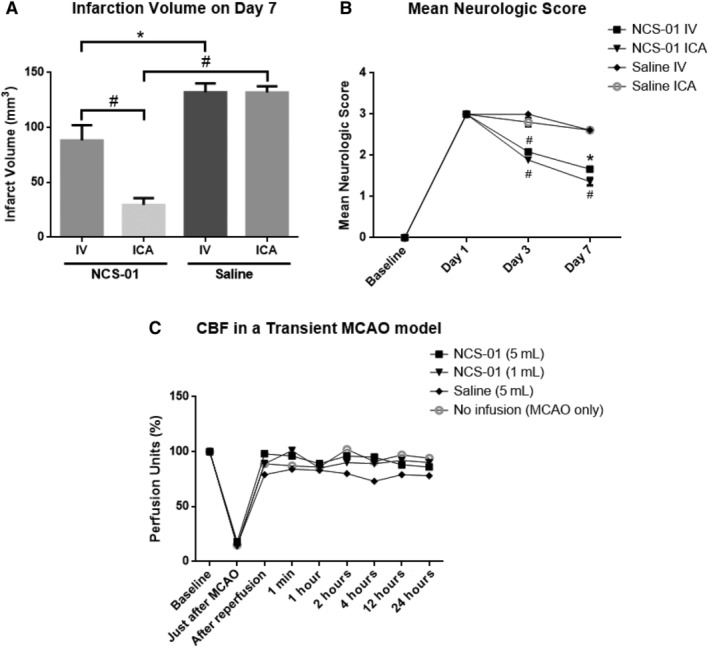
Intracarotid artery (ICA) transplants of NCS‐01 cells attenuate stroke‐induced behavioral and histological deficits. Route efficacy was measured by comparing changes in infarct volume and neurological deficit after administration of either NCS‐01 cells or saline (A, B). Both routes of NCS‐01 cell administration decreased both infarction (*P < .05, #*P* < .0001) and neurological deficit scores (*P < .05, #*P* < .0001 vs saline) as compared to saline, but a difference was observed between ICA and IV delivery only in measures of infarct volume. In this case, ICA‐administration was more effective than IV‐administration. No difference was observed between routes for neurological deficit. In addition, two dosages of NCS‐01 cells, saline, or no infusion (MCAO only) displayed no substantial differences when using cerebral blood flow assessment as a measure of safety indicating that ICA NCS‐01 cells did not alter blood flow and was safe (albeit did not lead to overt embolism) (C)

IV‐delivered or ICA‐delivered NCS‐01 cells provided substantial neurologic and pathologic benefit as compared to saline when administered in rats with transient MCAO (Figure 2A,B). Furthermore, at 7 days poststroke, the ICA‐administered NCS‐01 cells reduced the infarct size almost twice as smaller as that seen with IV administration (*P* < .0001). Thus, ICA delivery was more effective than the IV route in ameliorating brain damage. In addition, neurological deficit was decreased with either route of NCS‐01 cell administrations compared to saline‐treated stroke rats. By day 7, NCS‐01 cell treatment groups scored approximately 40% lower than the saline groups (*P* < .05, *P* < .0001). This measure was unaffected by route type, presumably indicating that the clinical measurements may not always reflect enhanced neurological function in all affected brain regions. Taken together, these results suggest that ICA delivery of NCS‐01 cells produced better stroke outcomes than IV delivery, at least in one aspect of brain damage. In addition, these results provided evidence that the therapeutic effects of NCS‐01 cells seen in vitro translated to functional benefits in the in vivo stroke model.

#### 3.1.3 ICA infusion of NCS‐01 cells poses no greater risk than no treatment or placebo

After determining logistical factors such as dosage and route, it is important to address safety concerns when considering eventual clinical translation of NCS‐01 cells. To evaluate ICA administration safety, CBF assessment was conducted. Male rats (n = 6 per group) were subjected to 1 hour transient MCAO and divided into groups receiving ICA administration of 5 × 10^7^ NCS‐01 cells in 5 mL, 1 × 10^7^ NCS‐01 cells in 5 mL, 5 mL of saline, or no infusion (MCAO only). The CBF was measured by Doppler at baseline (before MCAO), just after MCAO, after reperfusion, and at 1 minute, 1 hour, 2 hours, 4 hours, 12 hours, and 24 hours post dosing. Perfusion units were calculated and were displayed as percentages. CBF was not decreased by ICA administration of 1 or 5 mL of NCS‐01 cells in the stroke model when compared to the no infusion group and the saline group (Figure 2C). This suggests that ICA administration of NCS‐01 cells may be just as safe as no infusion, when operationalized by CBF assessment.

#### 3.1.4 Doses as low as 7.5 × 10^5^ NCS‐01 cells substantially reduce infarct volume and improve neurological function

**Figure 3:**
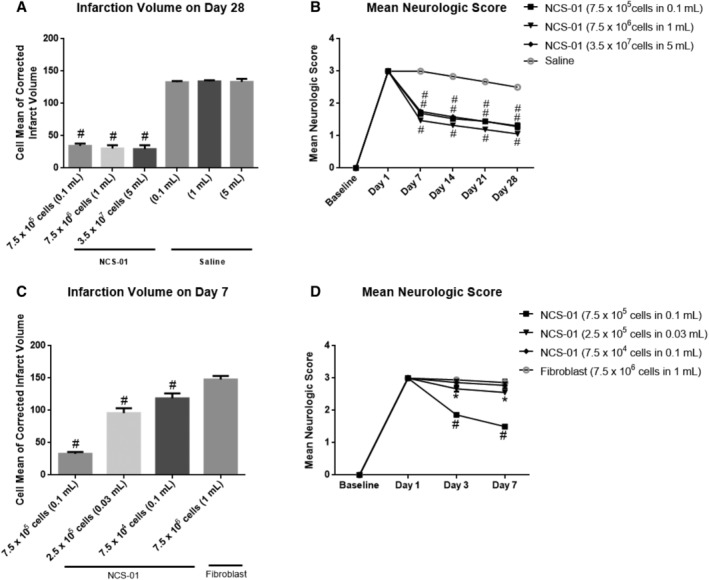
A mid‐range dose of NCS‐01 cells offers the greatest value while maximizing effects. Having established efficacy, route, and safety, multiple measures manipulating dosage were conducted. High doses, ranging from 7.5 × 10^5^ to 3.5 × 10^7^ NCS‐01 cells, yielded roughly equivalent effects on both infarct volumes and longitudinal (28 days) neurological deficit (*P < .05, #*P* < .0001 vs saline) (A, B). Following these results, a lower set of dosages was tested, ranging from 7.5 × 10^4^ to 7.5 × 10^5^ NCS‐01 cells (*P < .05, #*P* < .0001 vs fibroblast) (C, D). Among these, 7.5 × 10^5^ NCS‐01 cells yielded the best results for both reduction of infarction volume and longitudinal (7 days) neurological deficit, although lower doses still exerted partial therapeutic effects when compared to fibroblast placebo

Based on the previous measures, it was determined that ICA administration is the better route than IV to deliver more cells to the target lesion. This measure also found a benefit of NCS‐01 cells for neurological function and infarction volume in MCAO rats. To further advance these findings, cell dosages were evaluated using the ICA administration. In the first investigation, groups of six rats were subjected to 1 hour transient MCAO and were given 7.5 × 10^5^, 7.5 × 10^6^, or 3.75 × 10^7^ NCS‐01 cells. To avoid confounding NCS‐01 cell dose with NCS‐01 cell concentration effects, cells were administered as a fixed cell concentration of 7.5 × 10^6^ cells/mL. Thus, the administration volumes were 0.1, 1.0, or 5.0 mL, respectively. Groups treated with NCS‐01 cells exhibited reduced infarct volumes that were less than a quarter of the size of the saline groups (*P* < .0001) (Figure 3A,B). Additionally, by day 28, neurological deficit scores of groups treated with NCS‐01 cells were approximately reduced by half compared to the scores of the saline groups (*P* < .0001). No significant differences were observed between the three NCS‐01 cell treatment groups for either infarct size or neurological function in this model. More importantly, a dose of 3.75 × 10^7^ NCS‐01 cells was well tolerated in rats via the ICA route up to 28 days post‐MCAO. These results provided further evidence of the ameliorative effects of NCS‐01 cells in vivo and suggest that dosages as low as 7.5 × 10^5^ cells could provide therapeutic effects.

To further advance the application of NCS‐01 cells to the clinic, a second experiment was conducted to establish a minimum effective in vivo dose. Various amounts of NCS‐01 cells as well as fibroblast cells were used to reduce infarct volume. Four groups of six rats were subjected to 1 hour transient MCAO and were then infused with either 7.5 × 10^5^ NCS‐01 cells in 0.1 mL, 2.5 × 10^5^ NCS‐01 cells in 0.03 mL, 7.5 × 10^4^ NCS‐01 cells in 0.1 mL, or 7.5 × 10^6^ rat fibroblast cells in 1 mL. Efficacy varied according to dosage (Figure 3C,D). All NCS‐01 cell‐treated groups displayed significantly lower infarct volumes when compared to the fibroblast group (*P* < .0001). For neurological function, stroke animals that received NCS‐01 cell dosages of 2.5 × 10^5^ cells and 7.5 × 10^5^ cells exhibited significantly lower deficit scores than the fibroblast group (*P* < .05, *P* < .0001). Additionally, the 7.5 × 10^5^ NCS‐01 cells in 0.1 mL dosage were far more effective than the other two NCS‐01 cell dosages for both reducing infarct volume and improving neurological function. Thus, 7.5 × 10^5^ NCS‐01 cells in 0.1 mL could anchor further studies as a standard minimum dosage for stroke models.

### 3.2 Study 2

#### 3.2.1 NCS‐01 cell treatment improves stroke outcomes after permanent MCAO, but more effectively promotes functional benefits in transient MCAO

**Figure 4:**
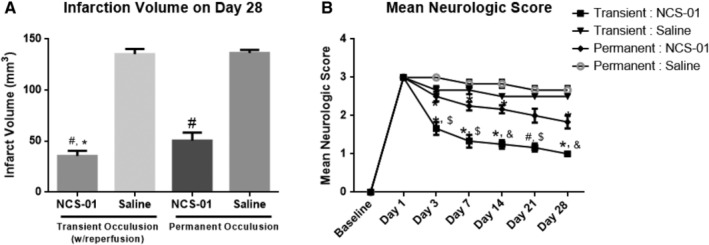
NCS‐01 cells confer therapeutic effects in both transient and permanent middle cerebral artery occlusion (MCAO) models, but more potently improve functional outcomes in ischemic‐reperfused stroke animals. Quantitative analysis of the infarction volume after 28 days in saline and NCS‐01 cells treated groups for transient and permanent occlusion. NCS‐01 cells decreased infarction volume nearly threefold compared to saline treatment. NCS‐01 cell treatment revealed a greater reduction in infarction volume for the transient occlusion than the permanent occlusion condition (*P < .001 vs NCS‐01 permanent, #*P* < .0001 vs saline) (A). Mean neurological score across 28 days for saline and NCS‐01 cell treated groups for transient and permanent occlusion. NCS‐01 cells improved neurological function in both the transient and permanent conditions, with a gradual increase across the 28 days (*P < .05, #*P* < .0001 vs saline, $*P* < .01, &*P* < .001 vs NCS‐01 permanent) (B). NCS‐01 cell treatment preferentially promoted greater histological and neurological improvements in animals exposed to transient MCAO than those subjected to permanent MCAO

To optimize the clinical target, it is necessary to examine the functional benefits of NCS‐01 cells in different disease conditions. Thus, the therapeutic effects of ICA‐delivered NCS‐01 cells were compared between transient MCAO and permanent MCAO (n = 6 per group). At 24 hours postocclusion, both conditions received either 1 mL saline (placebo) or 7.5 × 10^6^ NCS‐01 cells in 1 mL. Overall, results show behavioral and histological improvement in both MCAO paradigms after 28 days. NCS‐01 cells reduced infarct volume by approximately 60% to 70% (*P* < .0001) (Figure 4A). Additionally, infarct volume was more greatly reduced in the transient MCAO than in the permanent MCAO (*P* < .001). For neurological deficit score, the benefit was two‐ to threefold greater (depending mainly on the poststroke observation time) with ICA NCS‐01 cells in the transient MCAO than in the permanent MCAO (*P* < .01, *P* < .001). The time course of neurological deficit showed steady improvement over the 28 days poststroke, averaging between 11% lower during this period for permanent + saline treated animals to 67% lower for transient + NCS‐01 cell‐treated animals (Figure 4B). Thus, under these pathological conditions, NCS‐01 cell treatment was more effective in transient MCAO than permanent MCAO, although it still had some ameliorative effect in permanent MCAO. It should be noted that the transient occlusion model mimics the situation in which a stroke patient experiences successful revascularization procedure, such as tPA treatment or clot removal via thrombectomy. The permanent occlusion mimics the situation in which a stroke patient neither received tPA nor underwent thrombectomy, possibly because the 4.5 hours time window had elapsed. This occurs in over 90% of all stroke cases.^42^

#### 3.2.2 NCS‐01 cell treatment remains effective even when initiated at 1 week post‐MCAO, but most effective when started at 3 days or earlier

Another critical area that must be examined before clinical translation is the optimal therapeutic window. Accordingly, three experiments assessed the effect of the timing of cell delivery in the transient MCAO model using the same dose of cells (7.5 × 10^6^ cells in 1 mL) as described above, but with treatment initiation ranging from 3 hours to 1 week post‐transient MCAO. Rats were treated with NCS‐01 cells (n = 6‐7) or saline (n = 3) and were followed for up to 28 days. The first experiment examined the timing of cell delivery between 3 and 24 hours poststroke. The placebo group was dosed with 1 mL saline at 24 hours post‐MCAO. All animals were assessed for neurological function up to day 7 and were sacrificed on day 7. The second experiment aimed to examine a lengthened time window by comparing the timing of cell delivery at 24 hours to 1 week (7 days) post‐transient MCAO. Placebo groups were dosed with saline at 24 hours or 7 days poststroke. All animals were assessed for neurological function up to day 28 and were sacrificed on day 28. Finally, the third experiment was conducted to further investigate the therapeutic window between day 1 and day 7. Rats subjected to 1 hour transient MCAO were administered either 1 mL saline at 24 hours or 1 week post‐MCAO or 7.5 × 10^6^ NCS‐01 cells in 1 mL at 1, 3, 5, or 7 days post‐MCAO. All animals were assessed for neurological function up to day 28 and were sacrificed on day 28.

**Figure 5:**
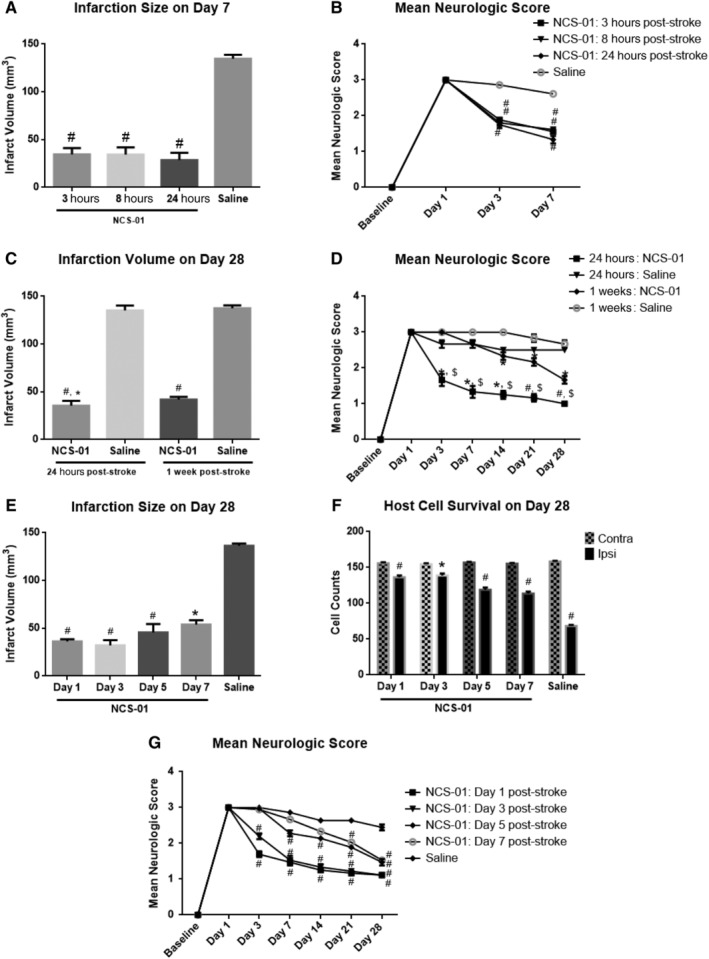
NCS‐01 cells remain a viable treatment option days after stroke. Quantitative analysis of the infarct volume across NCS‐01 cell treatment groups. A reduction in infarction size was observed in all NCS‐01 cell groups compared to the saline group. NCS‐01 cell treatment was most effective in decreasing infarct volume when administered 1 or 3 days poststroke, but infarct reduction was still determined at least up to 7 days post‐middle cerebral artery occlusion (MCAO) (*P < .05, #*P* < .0001 vs saline) (A, E) (*P < .05 vs NCS‐01 1 week, #*P* < .0001 vs saline) (C). Scores reflecting neurological function in saline and NCS‐01 cell treatment groups. NCS‐01 cells improved neurological scores across all treatment groups compared to saline, with the most improvement at administration 1 day post‐MCAO (*P < .05, #*P* < .0001 vs saline, $*P* < .0001 vs NCS‐01 1 week) (B, D, G). Host cell survival in the ipsilateral (ipsi) and contralateral (contra) peri‐infarct area across NCS‐01 cell treatment groups at 28 days post‐MCAO. All NCS‐01 cell groups exhibited increased cell survival in their ipsilateral peri‐infarct area (closer to the cell counts in their contralateral peri‐infarct area) compared to the saline group (*P < .05, #*P* < .0001 vs each contra) (F). Initiation of NCS‐01 cell treatment beyond 3 days following stroke moderated the functional outcomes

All NCS‐01 cell treatment groups significantly improved over the saline groups on all test outcomes by day 28 or even at earlier time points (Figure 5). Infarct volumes (Figure 5A,C,E) varied between approximately 60% and 80% reduction in all NCS‐01 cell treatment groups compared to saline placebos (*P* < .05, *P* < .0001). The greatest reductions were found in rats treated with NCS‐01 cells at day 1 and day 3 poststroke (*P* < .0001). While still significant, infarct reduction was somewhat attenuated for rats treated with NCS‐01 cells more than 3 days post‐MCAO. A similar pattern of results was observed in neurological deficit scores (Figure 5B,D,G). Neurological function improved in all NCS‐01 cell treatment groups over the saline group, but this improvement was again slightly attenuated in rats treated more than 3 days post‐MCAO (*P* < .05, *P* < .0001). Lastly, the third experiment also measured host cell survival in the contralateral (not directly affected by MCAO) and ipsilateral (directly affected by MCAO) peri‐infarct area (Figure 5F). Again, all NCS‐01 cell treatment groups exhibited better host cell survival than the saline group, but this effect was attenuated for rats treated more than 3 days post‐MCAO. Taken together, the data showed that delivery of NCS‐01 cells ameliorated brain damage and neurological deficit, but that delaying treatment after 3 days post‐MCAO may not provide as much functional benefits. This time‐dependent therapeutic outcome of NCS‐01 cells is a significant finding as it suggests the ideal clinical target would likely be patients less than 3 days after onset of ischemic stroke.

### 3.3 Study 3

#### 3.3.1 Other MSCs (Li cells) and NCS‐01 cells display comparable therapeutic effects on host cell viability

**Figure 6:**
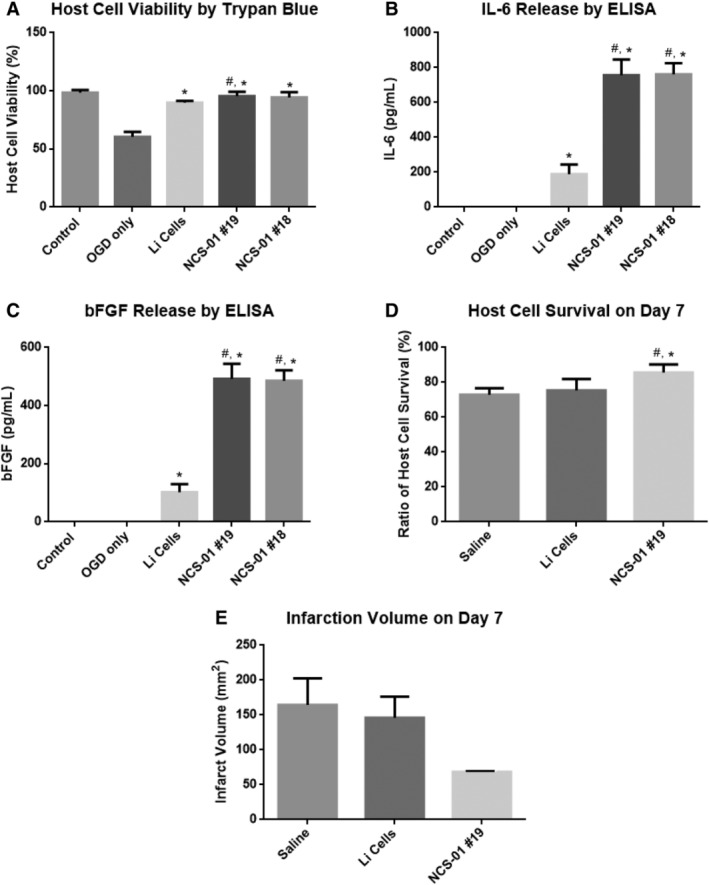
NCS‐01 cells enhance cell survival, reduce infarction volume, and secrete cytokines greater than Li cells in vitro and in vivo. Trypan blue assay revealed increased in vitro host cell viability by lot 18 of NCS‐01 cells, lot 19 of NCS‐01 cells, and Li cells following oxygen glucose deprivation (OGD) (*P < .001 vs OGD only, #*P* < .05 vs Li cells) (A) compared to ambient condition (control). Both lots of NCS‐01 cells expressed a near fourfold elevation in IL‐6 and bFGF over the Li cells (*P < .0001 vs OGD only, #*P* < .0001 vs Li cells) (B, C). Lot 19 of NCS‐01 cells increased in vivo host cell survival and dramatically reduced infarct volume by ~60% at 7 days poststroke compared to the Li cells (*P < .05 vs Li cells, #*P* < .0001 vs saline) (D, E)

In an effort to determine whether NCS‐01 cells performed equally or better than other MSCs, we initially embarked on in vitro tests of efficacy comparing NCS‐01 cells and Li MSCs, which were prepared as described elsewhere.^43^ Culture wells containing 4 × 10^4^ human neural progenitor cells were subjected to OGD, then lot 18 or lot 19 of NCS‐01 cells were added to the host cell wells. Both lots of NCS‐01 cells all and Li cells rescued against OGD‐induced human host cell death as measured by trypan blue assay (Figure 6A).

#### 3.3.2 NCS‐01 cells produce greater amounts of IL‐6 and bFGF in vitro

Additionally, potential mechanisms of action for the neurorestorative effects of NCS‐01 cells on cultures of primary rat cortical neurons and astrocytes were explored. In the brain, MSCs may secrete trophic factors and cytokines such as BDNF, β‐NGF, IGF‐1, VEGF, bFGF, and IL‐6. Since these trophic factors and cytokines have the potential to rescue cells against the deleterious effects of ischemic insult, a preliminary test was performed by assaying concentrations of these factors in the OGD model with commercially available ELISA kits. A 1:1 ratio of NCS‐01 cells to primary rat cortical neurons and astrocytes was used in the wells. Both lots of NCS‐01 cells and Li cells increased bFGF and IL‐6 (*P* < .0001) (Figure 6B,C), but none of the other tested trophic factors (BDNF, β‐NGF, IGF‐1, and VEGF) were detected. These findings suggest that IL‐6 and bFGF may be the mechanisms of action behind the amelioration observed after in vitro MSC treatment. Furthermore, NCS‐01 cells produced approximately four times as much IL‐6 and bFGF compared to Li cells (*P* < .0001), indicating that NCS‐01 cells exhibited distinct phenotypes and that these specific cytokines were closely associated with the more potent effects of NCS‐01 cells than Li cells. Additionally, NCS‐01 cell lot 18 and lot 19 produced identical results, providing quality assurance that the same optimized manufacturing process can produce the same cell population for treating stroke.

#### 3.3.3 NCS‐01 cells produce better therapeutic effects as assessed by host cell survival, infarction volume, and neurological function in vivo than Li cells

The preliminary results from the preceding in vitro experiment motivated us to pursue in vivo assessments. Rats (n = 6 per group) were subjected to transient MCAO, and then treated with either saline, Li cells, or lot 19 NCS‐01 cells. Animals were assessed for neurological function up to day 7 and were sacrificed on day 7. Treatment effects were assessed with host cell survival, infarction volume, and neurological deficit tests.

**Figure 7:**
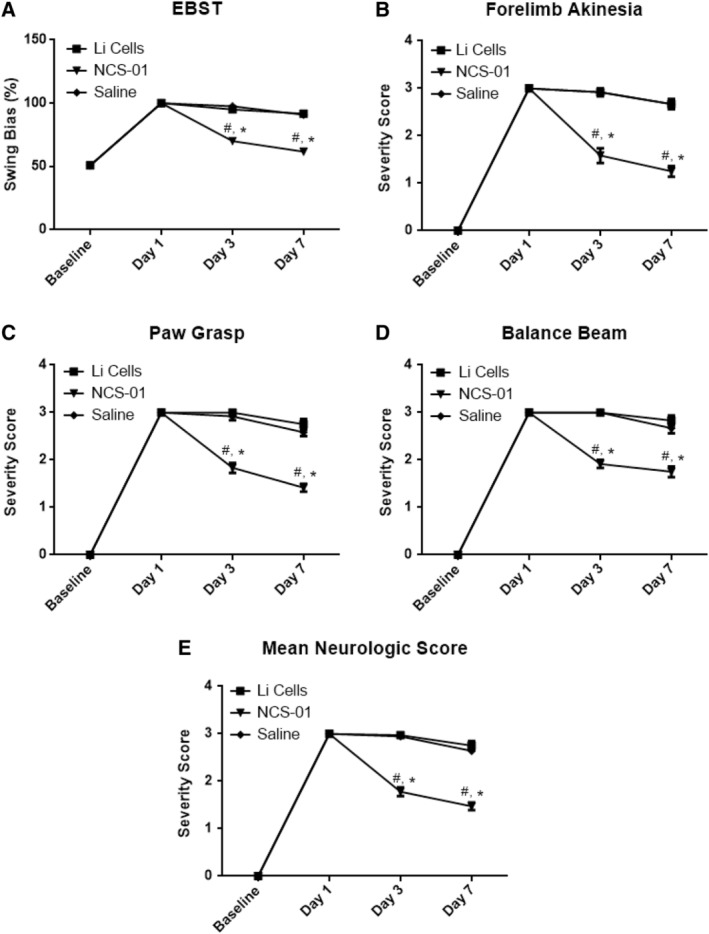
NCS‐01 cells improve behavioral and neurological outcomes better than Li cells and saline. Motor function evaluated by the Elevated Body Swing Test (EBST) (A), contralateral hind limb retraction (B), bilateral forepaw grasp (C), and beam walking ability (D) (*P < .0001 vs Li cells, #*P* < .0001 vs saline). NCS‐01 cell treatment displayed improved behavioral function compared to the Li cells or saline. Stroke rats treated with NCS‐01 cells demonstrated reduced neurological deficits, nearly half that compared to the Li cells and saline, across 7 days (*P < .0001 vs Li cells, #*P* < .0001 vs saline) (E)

NCS‐01 cells, but not Li cells, rescued against in vivo host cell death by ameliorating ischemia‐induced brain damage with consequent ~60% reduction in infarction (*P* < .0001) (Figure 6D,E). In addition, as a consequence of ameliorating ischemia‐induced brain damage, NCS‐01 cells reduced neurological deficit by half (*P* < .0001). Again, the same therapeutic effect was not seen with Li cells (Figure 7). Based on the observed in vivo functional and histological benefits of NCS‐01 cells over Li cells, this NCS‐01 cell population, defined by the selected manufacturing process, is a unique donor cell population for transplantation therapy. Combining these in vivo observations with the previous in vitro data, as well as the results of studies 1 and 2, presents a compelling case for declaring safety and efficacy of NCS‐01 cells in treating ischemic stroke.

#### 3.3.4 NCS‐01 cells may rescue primary rat cortical cells via filopodia formation

**Figure 8:**
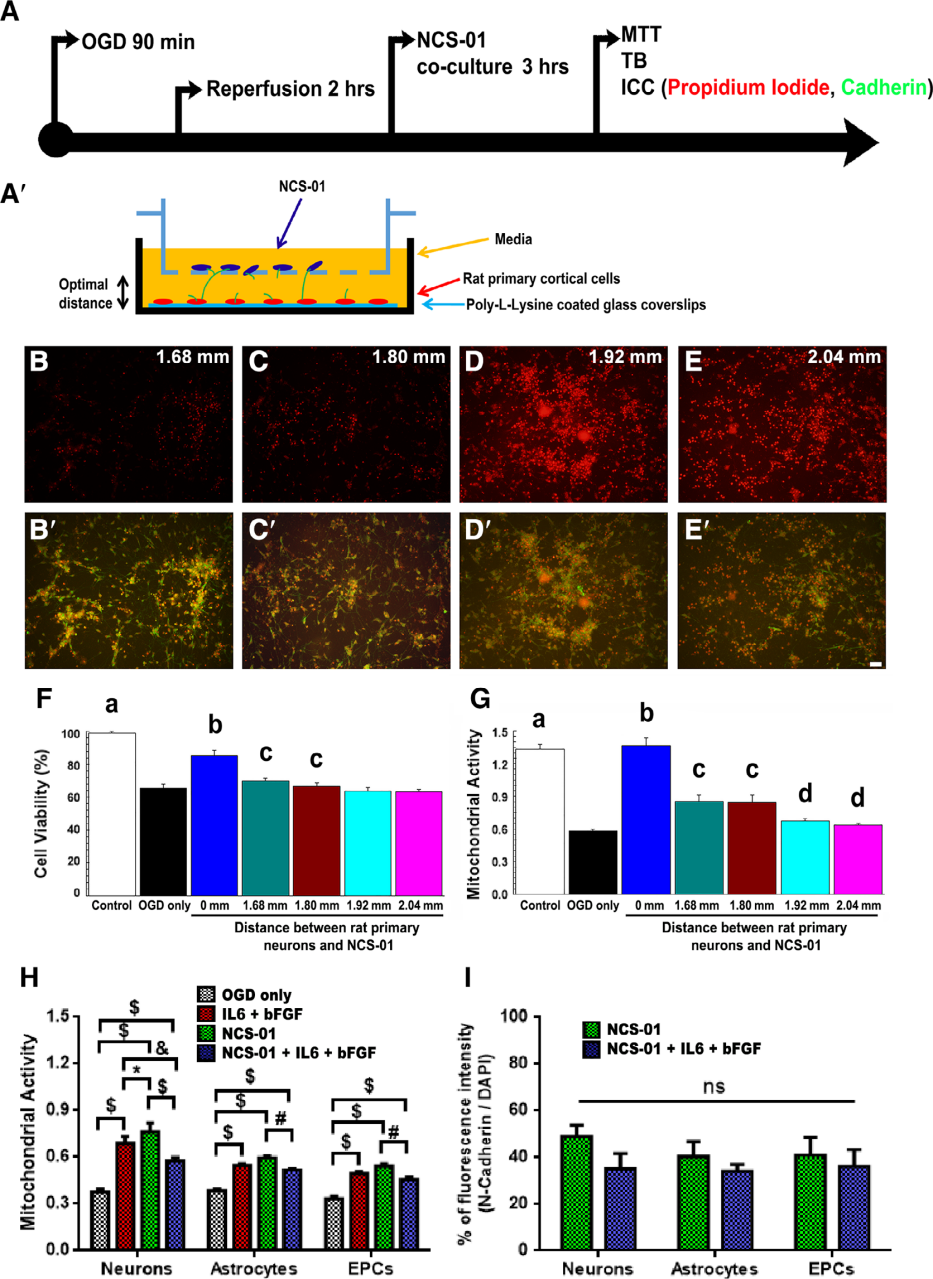
NCS‐01 cells, in part, employ filopodia‐mediated long distance rescue of cultured primary rat cortical cells against oxygen glucose deprivation (OGD). Primary rat cortical cells were subjected to OGD and reperfusion then cocultured with NCS‐01 cells at different distances, as depicted by procedural timeline and diagram (A and A′). Propidium iodide and N‐cadherin staining was conducted for the different distances: (B and B′) 1.68 mm, (C and C′) 1.80 mm, (D and D′) 1.92 mm, and (E and E′) 2.04 mm. Propidium iodide staining (red) reveals fewer dead primary rat cortical cells (indicated by red color) at closer distances than at farther distances between upper and lower chambers (B‐E). N‐cadherin staining (green) indicates the presence of filopodia extending from NCS‐01 cells (B′‐E′) Scale bar = 50 μm. Quantitative analysis of primary rat cortical neurons reveals that cell viability correlates inversely with increased distance between NCS‐01 cells and primary rat cortical neurons, when measured by trypan blue (TB) assay (F). Similarly, MTT assay reveals that greater mitochondrial activity correlates inversely with greater distance between NCS‐01 cells and primary rat cortical neurons (G). Significance depicted as a‐d at *P* < .05, a: greater than OGD only and all treatment groups; b: greater than all groups except control, non‐OGD group; c: greater than OGD only and 1.92 and 2.04 mm groups; d: greater than OGD only (F, G). Additionally, primary rat cortical neurons, primary rat astrocytes, and primary rat EPCs were subjected to OGD and reperfusion then treated with IL‐6 + bFGF and/or cocultured with NCS‐01 cells at 0.8 mm distance. MTT assay displays greatest mitochondrial activity in all cell types when cocultured with NCS‐01 cells only or treated with IL‐6 + bFGF only (*P < .05, #*P* < .01, &*P* < .001, $*P* < .0001) (H). Moreover, N‐cadherin staining demonstrates no significant difference in filopodia formation in the two cocultured groups and a similar pattern among different cell types (I)

Following OGD and reperfusion (Figure 8A), primary rat cortical cells were cocultured with NCS‐01 cells at different distances ranging from 0 mm (direct contact) to 2.04 mm (Figure 8A'). Cultures were stained with propidium iodide to reveal cell viability and N‐cadherin to detect filopodia (Figure 8B‐E'). Imaging revealed the presence of filopodia extending from NCS‐01 cells to ischemic cells. Quantitative analyses detected maximal (about 85% normal levels) rescue of cell viability and mitochondrial activity from direct contact of NCS‐01 cells with primary rat cortical cells compared to the cells exposed to OGD treated with “non‐stem cells” (Figure 8F,G). The two closer distances (1.68 and 1.80 mm), and, to a lesser degree, the two farther distances (1.92 and 2.04 mm), also significantly improved cell viability and mitochondrial activity compared to the non‐stem cell condition. Taken together, these results indicate direct cell‐to‐cell contact as optimally effective but suggest the potential to achieve equally robust ameliorative effects against OGD via indirect long distance rescue via filopodia formation (Supplemental Figure S2).

In order to probe the exact role of IL‐6, bFGF, and NCS‐01‐derived filopodia in the rescue of different types of host cells, an additional measure was performed by exposing primary cortical neurons, primary rat astrocytes, and primary rat EPCs to OGD and reperfusion and treating with cell media only (OGD only control), IL‐6 + bFGF, NCS‐01 cell coculture, and a combination of IL‐6 + bFGF and NCS‐01 cell coculture. MTT assay reveals that compared to OGD only, all treatments improved mitochondrial activity in a similar pattern among cell types, with the greatest activity in the groups treated with NCS‐01 cell coculture only and IL‐6 + bFGF only (Figure 8H). Neurons display the greatest recovery and are the only cell types to exhibit a slight but significant difference between IL‐6 + bFGF treatment only and NCS‐01 cell coculture only, with the latter performing slightly better than the former treatment (*P* < .05). There was no significant difference between IL‐6 + bFGF‐mediated and NCS‐01‐mediated improvements for astrocytes and EPCs. Interestingly, the combined treatment groups, while significantly improved compared to OGD only, performed substantially worse than either of their respective stand‐alone groups in every case (*P* < .01, *P* < .001, *P* < .0001), with the minor exception of astrocytes treated with IL‐6 + bFGF and those treated with combined, which did not differ significantly (*P* > .05). Furthermore, across all three neural cell lineages, N‐cadherin staining indicates 35% to 55% increments in fluorescence intensity in stand‐alone NCS‐01 cell coculture or combined treatment compared to OGD only, indicating filopodia formation accompanied the improved cell viability and mitochondrial activity in these treatments (Figure 8I). The observed increment in N‐cadherin staining did not significantly differ between NCS‐01 cell coculture only and combined treatment, suggesting similar robust filopodia formation in both treatments. Overall, these results bolster our claim that improvements in mitochondrial activity via IL‐6 and bFGF release and filopodia formation may be the primary modes of action of NCS‐01 cells' rescue of host cells.

## 4 DISCUSSION

The present study assessed the potential of NCS‐01 cells as a cell‐based therapy for stroke by conducting a large series of experiments distributed in three major translational studies. In the first study, we evaluated the efficacy, safety, and mechanism of action of NCS‐01 cells in standard in vitro and in vivo models of ischemic stroke. Initially, we showed that NCS‐01 cells rescue ischemic cells in a dose‐dependent manner in the OGD model. Consequently, we observed upregulated levels of bFGF and IL‐6 in the supernatant of cultured NCS‐01 cells, implicating therapeutic molecule secretion as a mechanism mediating the cells' functional effects. in vivo results showed that ICA cell delivery produced a significant dose‐dependent reduction in infarct size more effectively than IV, possibly due to its capacity to deliver more cells, and likely therapeutic molecules, into the ischemic brain. The effective dosage was 7.5 × 10^6^ cells in 1 mL. The ICA cell delivery route was safe as evidenced by no observable alterations in the CBF and absence of microembolic events.

The second study was designed to assess the disease condition (transient or permanent MCAO) and therapeutic window in the in vivo model to gain insights into the clinical target population. The results revealed that ICA delivered NCS‐01 cells ameliorated that stroke‐induced behavioral and histological impairments associated with both transient and permanent MCAO, but the functional improvements in the stroke animals with transient ischemia was two‐ to threefold greater than those with permanent occlusion, suggesting that this therapy would provide more beneficial effects in stroke patients who were successfully revascularized either by tPA or thrombectomy. The therapeutic window was then evaluated in the transient MCAO model by using the same optimal effective dose of cells (7.5 × 10^6^ cells in 1 mL) as described above but delivered at varying time points from 3 hours to 1‐week post‐MCAO. The data showed that the delivery of NCS‐01 cells within 3 days post‐MCAO appears to produce maximal therapeutic effects against MCAO, but that delaying treatment beyond 3 days post‐MCAO, although still effective, may not provide robust functional benefits. Altogether, these results suggest that the relatively early (less than 3 days) administration of NCS‐01 cells in transient MCAO corresponded to the most responsive stroke population to this cell therapy. However, even 1 week after transient MCAO or with permanent occlusion, NCS‐01 cells still produced meaningful functional recovery. Such wide therapeutic window, if at all an indication of the stroke prognosis, suggests that NCS‐01 cells may provide a clinically practical treatment option for individuals who could not avail of tPA and thrombectomy.

The third study was conducted to evaluate the neurorestorative properties of NCS‐01 cells compared to other MSCs (Li cells) employed for cell therapy in stroke. in vitro results showed that both cell types were able to rescue against OGD‐induced host cell death; however, NCS‐01 cells increased cytokine (IL‐6 and bFGF) release more than threefold compared to Li cells. This indicates that NCS‐01 cells phenotypically differ from Li cells, even though both are characterized as MSCs. More importantly, during the in vitro studies, two different lots of NCS‐01 cells produced identical results, hence equivalence of these two cell lots was achieved with the same optimized manufacturing process. Furthermore, comparison between neurons, astrocytes, and EPCs generally revealed similar patterns of NCS‐01 cell culture‐mediated and IL‐6 + bFGF‐mediated improvements in mitochondrial activity, supporting potential modes of action for NCS‐01's rescue of host cells in vitro. Subsequent in vivo studies demonstrated that at 24 hours postocclusion, the ICA infusion of NCS‐01 cells, but not Li cells, ameliorated brain infarction and improved neurological deficits. While Li cells may promote therapeutic effects at a higher dose, when using the same doses for both MSCs, NCS‐01 cells outperformed the Li cells in efficacy outcomes.

Finally, we observed for the first time a novel mechanism involving filopodia formation in stem cells under stroke conditions. That stem cells may propel cell processes in long distances toward the ischemic cells suggests the potential for transplanted cells to engraft in a conducive environment remote from the injured tissue, but still rescue ischemic cells. Filopodia formation has been previously observed in neuroprotective action by Rho kinase inhibition on organotypic hippocampal slices against in vitro ischemia.^44^ Similarly, overexpression of transmembrane glycoprotein CD44 in vitro promotes the elongation, spread, and number of filopodia of cultured neural precursor cells, while in vivo accelerates the transendothelial migration and facilitates the invasion of certain perivascular sites.^45^ Filopodia formation and cell motility, especially transendothelial migration, may be facilitated by adhesion molecules, such as Ninjurin 1,^46^ and transcription factors, including serum response factor.^47^ Understanding the putative role of these transmembrane glycoproteins, adhesion molecules, and regulatory factors may improve filopodia formation, as well as the resulting therapeutic outcome of NCS‐01 cells in stroke.

Stroke is one of the main causes of disability among adults worldwide, with risk factors such as aging, hypertension, hyperglycemia, diabetes mellitus, and obesity.^48^, ^49^ The only FDA‐approved drug is tPA with limited treatment window and high risk of hemorrhagic transformation; hence, there is a significant need for finding a novel treatment for stroke. Recently, stem cell therapy has emerged as a promising experimental treatment, even reaching clinical trials, for neurological diseases, including stroke,^49^, ^50^ possibly due to the grafted cells' bystander effects such as secretion of growth factors and cytokines associated with neurogenesis, angiogenesis, vasculogenesis, and mitochondrial repair.^48^, ^49^, ^51^, ^52^ Here, we demonstrated the appealing therapeutic properties of NCS‐01 cells, namely cell secretion of bFGF and IL‐6, and filopodia extension in rescuing ischemic cells.

The present study revealed many novel observations regarding the use of NCS‐01 cells for stem cell therapy in stroke. First, we showed the safety and efficacy profile of NCS‐01 cells, supporting the cells' potential use as a therapeutic option for stroke. NCS‐01 cells rescued cell death, decreased infarct size, and improved neurological outcomes after stroke. Second, we identified the minimal optimal dosage and demonstrated that ICA route of delivery is safe and effective. We detected that there was no change in blood flow in association with the ICA delivery. Furthermore, ICA enhanced the delivery of NCS‐01 cells to the brain at the same time lowering the effective dose, further improving the safety profile of the cells. Third, we demonstrated that NCS‐01 cells are not only effective at reducing infarct volume and improving neurological score at early therapeutic window (hours after stroke) but also can be extended into days poststroke. The observed functional benefits persisted long after the administration of NCS‐01 cells, indicating robust and stable therapeutic outcomes. This set of data supports the notion that NCS‐01 cells could be used to treat a large number of stroke patients ranging from time of admission to days during poststroke stabilization. We also demonstrated that NCS‐01 cells are beneficial for either transient or permanent occlusion MCAO paradigms. This supports the use of NCS‐01 cells even for stroke patients who might have missed the opportunity to receive revascularization procedures such as tPA or endovascular interventions. Finally, we provided mechanism‐based evidence that NCS‐01 cells released significantly higher levels of cytokines bFGF and IL‐6, clearly distinguishing them from routinely harvested MSCs. The pioneering observation of formation of long processes of filopodia extending from NCS‐01 cells toward ischemic cells opens new avenues of research for engineering stem cells designed to propel cell processes to bridge the gap between normal and pathologic (ie, ischemic) tissues. Because the ischemic tissue limits cell survival of exogenous and endogenous stem cells, the neighboring tissues adjacent to the core or peri‐infarct area may be conducive for stem cell survival. Although direct cell‐to‐cell contact between stem cells and ischemic cells was shown optimal, NCS‐01 cells' potential to form filopodia over long distances from the spared tissue to the ischemic area may represent a novel repair mechanism allowing graft‐induced remote regeneration of ischemic cells. Taken together, NCS‐01 cells stand as potent transplantable cells with robust and innovative neuroprotective properties for stroke therapy.

Despite our extensive study designs and findings, there are certain limitations in the present study. In particular, future studies may consider using large animal models, such as porcine and/or nonhuman primate, to better mimic the human clinical pathologies (ie, white matter injury) and to further elucidate the current optimal dose, treatment timing, and route of administration of NCS‐01 cells. In addition, since NCS‐01 cells are derived from human cells and would be used for humans, the current human‐to‐rat paradigm may not fully capture the envisioned human‐to‐human clinical product. Another limitation is our relatively young stroke animals did not exhibit comorbidities associated with stroke, warranting the need to test this cell therapy in a stroke model with comorbidity factors such as aging and hypertension.^53^ Furthermore, while the secretion of bFGF and IL‐6 coupled with the filopodia formation accompanied the therapeutic effects of NCS‐01 cells, further manipulation of these phenotypic functions (eg, upregulating or downregulating cytokine release, and facilitating or inhibiting filopodia formation) may reveal more detailed brain repair machinery of this cell therapy.^54^ Another limitation is suggested by the marked disparity between the size of the effects observed in vivo and in vitro. That the in vitro effects paled in comparison with the in vivo effects when analyzing individual cell lineages may be partially explained by the overall effects of NCS‐01 on multiple cell phenotypes (neurons, astrocytes, and EPCs), reminiscent of the in vivo neurovascular unit, indicating a multipronged therapeutic mechanism. Moreover, that the combination of exogenously administered IL‐6 and bFGF and NCS‐01 cell transplantation did not lead to additive or synergistic effects, and instead tapered the functional improvements compared to stand‐alone treatments may be due to redundant therapeutic signaling pathways solicited by NCS‐01 cells, IL‐6, and bFGF, suggesting that overloading the system with mere neuroregenerative cytokines does not necessarily equate to enhanced therapeutic outcomes. NCS‐01 cells' ability to interact with the microenvironment niche, via the cells' filopodia formation, may allow a reciprocal and regulated modulation of the cytokine secretion toward neuroregeneration. Finally, although solid safety record of MSCs is documented in hematological indications, additional studies may be warranted to address the cells' high proliferative capacity.^50^, ^54^, ^55^ Notwithstanding these scientific limitations, these translational studies formed the key material of an IND package that was deemed by the FDA as sufficient to grant approval in July 2019 to proceed with the clinical trial of NCS‐01 cells in ischemic stroke patients.

In conclusion, the foregoing studies provide insights into the efficacy and mechanisms of NCS‐01 cells in treatment of ischemic injury in both in vitro and in vivo rat models (Graphical Abstract). We demonstrated that NCS‐01 cells were able to effectively improve motor and neurological behaviors by significantly reducing the infarct area and cell loss in the neighboring peri‐infarct region, possibly via secretion of therapeutic molecules and our pioneering observation of filopodia formation in stem cells. Although our study showed that NCS‐01 cells secreted bFGF and IL‐6 in the present OGD in vitro condition, additional cytokines and cell‐surviving factors may be secreted since NCS‐01 cells likely will adjust their secretory among other phenotypic functions in response to the dynamic brain microenvironment under in vivo stroke setting. Additionally, direct cell‐to‐cell contact, including filopodia interacting with other protrusive cell membranes may be required for the observed therapeutic activities. The ICA infusion appeared to improve cell delivery to the ischemic brain due to its capacity to directly deliver the highest number of cells to the lesion area without losing a high percentage of cell concentration to other circulatory areas. We further demonstrated that NCS‐01 cells maintained a therapeutically active state under a dose‐dependent mechanism both in vitro and in vivo paradigms. The therapeutic window indicated that even though the best time to administer the NCS‐01 cells was within 3 days post‐MCAO, beneficial effects were still recognized when administered at 1 week after MCAO. Compared with other MSCs, NCS‐01 cells ameliorated both neurostructural and functional deficits after stroke through a broad therapeutic window. Collectively, these translational observations provided solid lab‐to‐clinic evidence supporting NCS‐01 cell therapy for stroke in the clinical setting.

## CONFLICT OF INTEREST

Y.K. declared employment and stock ownership with the University of South Florida. T.N.C. declared consultant/advisory role and patent holder with KM Pharmaceutical Consulting LLC. M.K. declared consultant consultant/advisory role, patent holder and stock ownership in KM Pharmaceutical Consulting LLC. C.V.B. declared employment with the University of South Florida; patent holder with Sanbio Inc, Athersys, Saneron, Astellas; consultant/advisory role with KMPHC, Astellas, Asterias, Chiesi Pharmaceuticals, Sanbio Inc,; research funding from Astellas, Asterias, International Stem Cell Corp, Saneron, NIH and stock ownership with Sanbio Inc. The other authors declared no potential conflicts of interest.

## AUTHOR CONTRIBUTIONS

Y.K., J.‐Y.L., N.T., J.P.T., T.L., E.R., S.‐J.Y.: experimental work, collection and/or assembly of data, data analysis and interpretation, manuscript writing; B.B.: experimental work, collection and/or assembly of data, data analysis and interpretation, manuscript writing, collection and/or assembly of related data, data analysis; S.C., A.B.C., C.K.: manuscript writing, collection and/or assembly of related data, data analysis; T.N.C., M.K.: data analysis and interpretation, manuscript writing, conception and design, project supervision, final editing and manuscript oversight; C.V.B.: experimental work, collection and/or assembly of data, data analysis and interpretation, manuscript writing, conception and design, project supervision, final editing and manuscript oversight.

## Supporting information


**Supplemental Figure 1 NCS‐01 cells exhibit robust viability in vitro but only modest engraftment post‐transplantation**. Following thawing of cryopreserved NCS‐01 cells (Progenitor Cell Therapy, US), at least 85% cell viability was obtained, with cells expressing human specific mitochondria counterstained with the nuclei marker DAPI (A). ICA‐delivered NCS‐01 cells successfully reached the ischemic cortical and striatal penumbra, but only survived modestly at around 3 hours after transplantation (B, magnified in B′) and at day 1 post‐transplantation (C, magnified in C′), and almost non‐detectable at day 3 post‐transplantation (D, magnified in D’). Despite the poor survival and low engraftment of NCS‐01 cells, host cell sparing appears to increase in the ischemia penumbra as evidenced by higher number of DAPI‐stained nuclei at day 3 (D) compared to earlier time points post‐transplantation (B, C). Red: human mitochondria; Blue: DAPI. Scale bar = 10X in Panels A‐D, and 20X in Panels B′‐D’.

**Supplemental Figure 2 NCS‐01 cells display filopodia formation**. When exposed to OGD‐conditioned medium, NCS‐01 cells exhibit filopodia at 1 hour (A), 2 hours (B), and 3 hours (C), which seem to become elongated over time. Following transplantation in stroke brain, there is evidence that NCS‐01 cells also show filopodia formation at 3 hours (D) and day 1 post‐transplantation (E). Scale bar = 50 μm.

## Data Availability

The data that support the findings of this study are available from the corresponding author upon reasonable request.
