# Supplementary figures and images for: Translating intracarotid artery transplantation of bone marrow‐derived NCS‐01 cells for ischemic stroke: Behavioral and histological readouts and mechanistic insights into stem cell therapy
Source: Stem Cells Transl Med. 2019 Nov 18;9(2):203–20. doi: 10.1002/sctm.19-0229 (PMC6988762; doi:10.1002/sctm.19-0229)

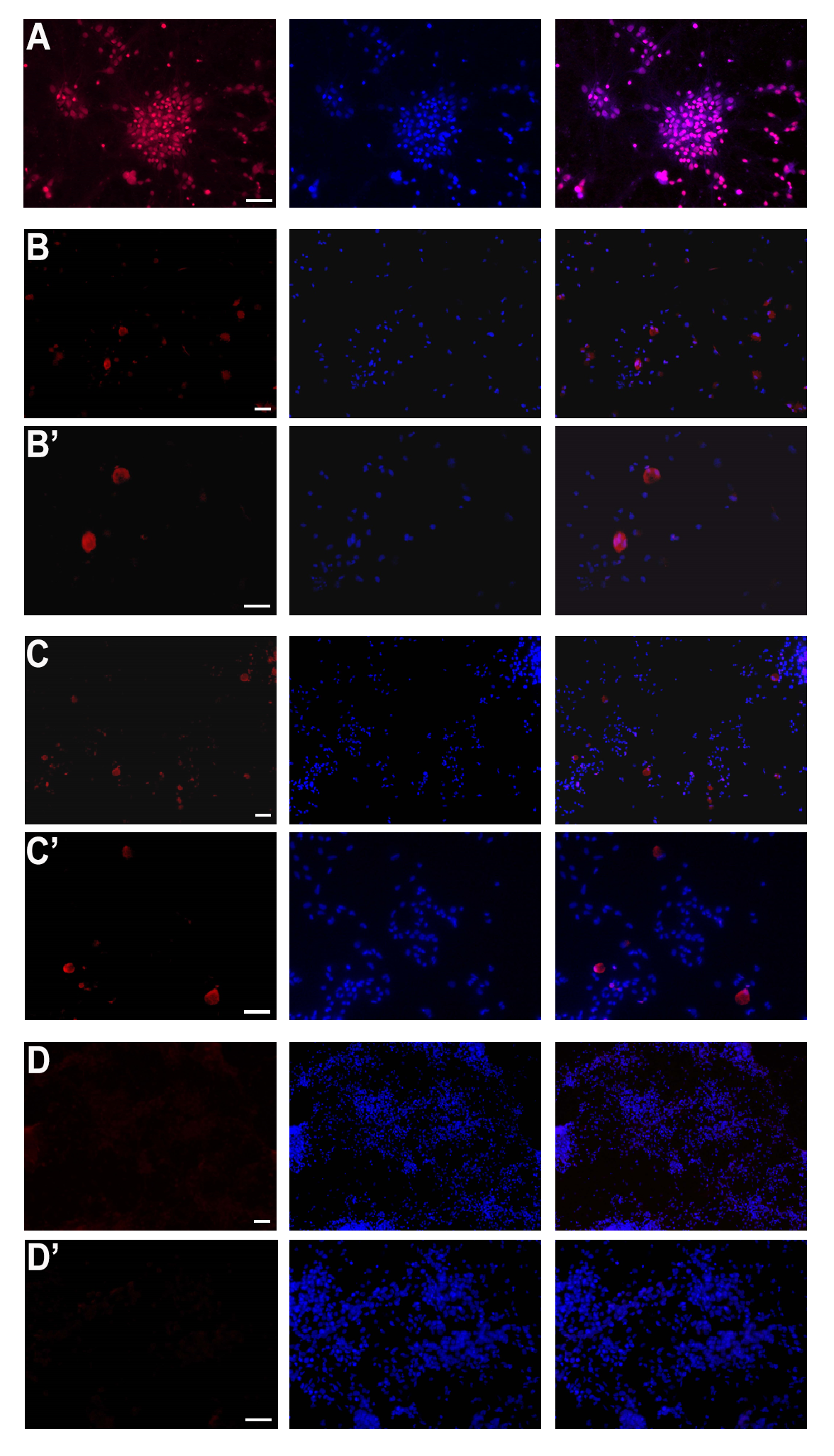

Supplement: Supplementary file 1 — Supplemental Figure 1 NCS‐01 cells exhibit robust viability in vitro but only modest engraftment post‐transplantation. Following thawing of cryopreserved NCS‐01 cells (Progenitor Cell Therapy, US), at least 85% cell viability was obtained, with cells expressing human specific mitochondria counterstained with the nuclei marker DAPI (A). ICA‐delivered NCS‐01 cells successfully reached the ischemic cortical and striatal penumbra, but only survived modestly at around 3 hours after transplantation (B, magnified in B′) and at day 1 post‐transplantation (C, magnified in C′), and almost non‐detectable at day 3 post‐transplantation (D, magnified in D’). Despite the poor survival and low engraftment of NCS‐01 cells, host cell sparing appears to increase in the ischemia penumbra as evidenced by higher number of DAPI‐stained nuclei at day 3 (D) compared to earlier time points post‐transplantation (B, C). Red: human mitochondria; Blue: DAPI. Scale bar = 10X in Panels A‐D, and 20X in Panels B′‐D’. [file SCT3-9-203-s001.tif]

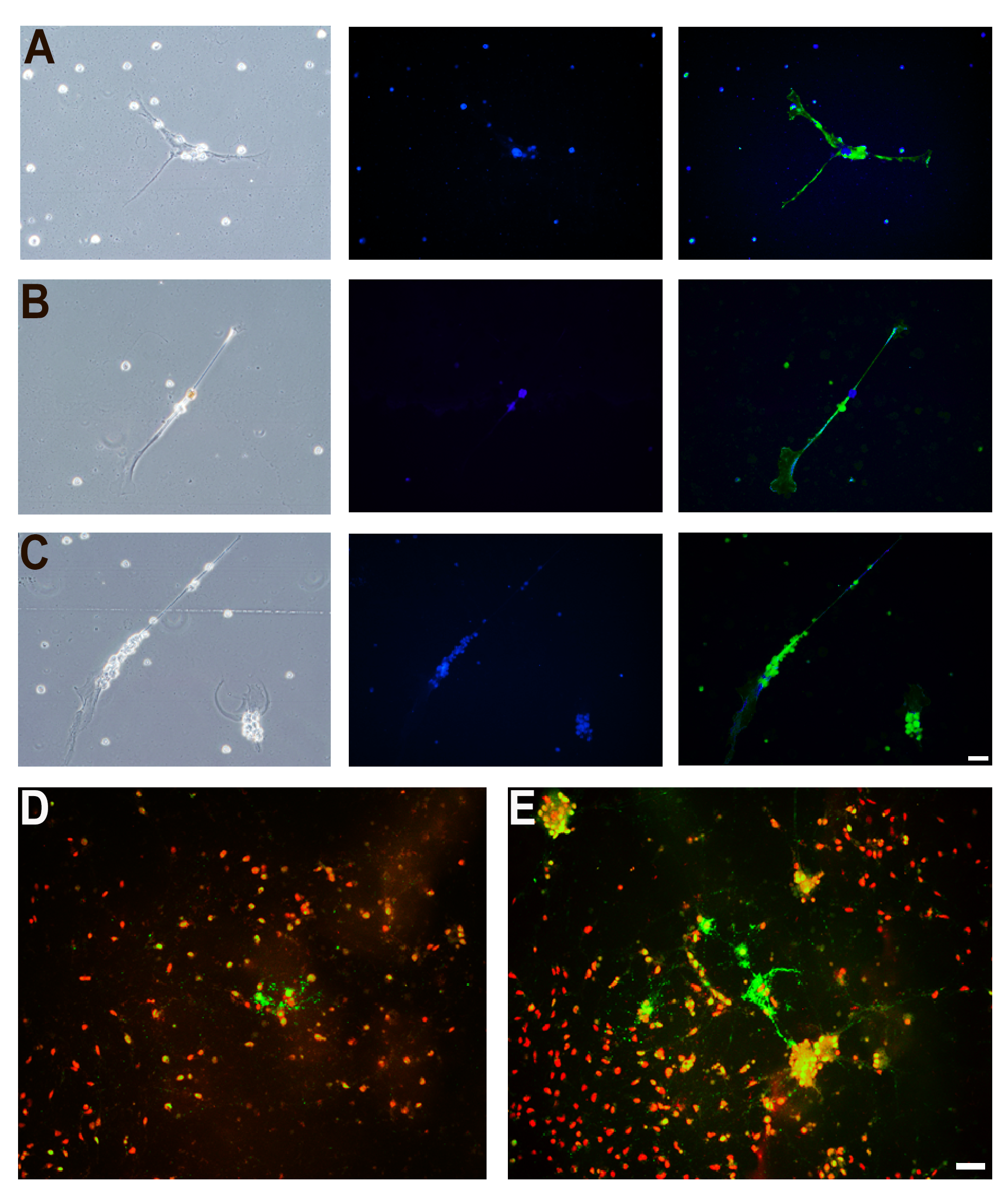

Supplement: Supplementary file 2 — Supplemental Figure 2 NCS‐01 cells display filopodia formation. When exposed to OGD‐conditioned medium, NCS‐01 cells exhibit filopodia at 1 hour (A), 2 hours (B), and 3 hours (C), which seem to become elongated over time. Following transplantation in stroke brain, there is evidence that NCS‐01 cells also show filopodia formation at 3 hours (D) and day 1 post‐transplantation (E). Scale bar = 50 μm. [file SCT3-9-203-s002.tif]
